# Effects of apnoea training on aerobic and anaerobic performance: A systematic review and meta-analysis

**DOI:** 10.3389/fphys.2022.964144

**Published:** 2022-09-27

**Authors:** Francisco de Asís-Fernández, Daniel Sereno, Anthony P. Turner, Fernando González-Mohíno, José María González-Ravé

**Affiliations:** ^1^ Departamento de Fisioterapia, Facultad de Ciencias de la Salud, Centro Superior de Estudios Universitarios La Salle, Universidad Autónoma de Madrid, Madrid, Spain; ^2^ Breatherapy Research Group, Instituto de Neurociencias y Ciencias del Movimiento (INCIMOV), Centro Superior de Estudios Universitarios La Salle, Universidad Autónoma de Madrid, Madrid, Spain; ^3^ Sports Training Laboratory, Faculty of Sports Sciences, Universidad de Castilla-La Mancha, Toledo, Spain; ^4^ Sport, Physical Education and Health Sciences, University of Edinburgh, Edinburgh, United Kingdom; ^5^ Facultad de Ciencias de la Vida y de la Naturaleza, Universidad Nebrija, Madrid, Spain

**Keywords:** breath-hold, sports performance, lactate, VO_2_max, endurance

## Abstract

**Background** Trained breath-hold divers have shown physiological adaptations that might improve athletes’ aerobic and anaerobic performance.

**Objective** This study aimed to systematically review the scientific literature and perform a meta-analysis to assess the effects of voluntary apnoea training on markers of anaerobic and aerobic performance, such as blood lactate and VO_2max_.

**Methods** A literature search on three databases (Web of Science, PubMed and SCOPUS) was conducted in March 2022. The inclusion criteria were 1) peer-reviewed journal publication; 2) clinical trials; 3) healthy humans; 4) effects of apnoea training; 5) variables included markers of aerobic or anaerobic performance, such as lactate and VO_2max_.

**Results** 545 manuscripts were identified following database examination. Only seven studies met the inclusion criteria and were, therefore, included in the meta-analysis. 126 participants were allocated to either voluntary apnoea training (ApT; *n* = 64) or normal breathing (NB; *n* = 63). Meta-analysis on the included studies demonstrated that ApT increased the peak blood lactate concentration more than NB (MD = 1.89 mmol*L^−1^ [95% CI 1.05, 2.73], z = 4.40, *p* < 0.0001). In contrast, there were no statistically significant effects of ApT on VO_2max_ (MD = 0.89 ml*kg^−1^*min^−1^ [95% CI −1.23, 3.01], z = 0.82, *p* = 0.41).

**Conclusion** ApT might be an alternative strategy to enhace anaerobic performance associated with increased maximum blood lactate; however, we did not find evidence of ApT effects on physiological aerobic markers, such as VO_2max_.

**Systematic Review Registration**: [PRISMA], identifier [registration number].

## Key points

- Results suggest that apnoea training might increase peak blood lactate concentration, potentially related to improved performance in sports requiring high anaerobic capacity, assuming an increased lactate tolerance. Further evidence is required to analyze potential benefits of apnoea training on maximal aerobic capacity.
- It is possible to generate severe hypoxaemia, increased lactate concentration and a greater glycolytic activity without being placed in a hypoxic environment, by using apnoea training at low lung volumes, eliciting faster hypoxaemia.
- Repeated apnoea training seems to be the most optimal method for use in athletes unfamiliar with apnoea training, as it is necessary to train an individual’s psychological tolerance of maximal apnoea.

## Introduction

In a breath-hold exercise, a succession of mechanisms elicit a range of physiological responses. First, even in dry conditions, the so-called diving reflex (Costalat, 2015) triggers a bradycardia powerful enough to nullify the exercise-induced increase in Heart Rate (HR) produced by breath-hold diving exercise (Breskovic, 2011). The diving response also elicits a centralization of blood flow by increased peripheral vasoconstriction (Andersson, 2009), as a consequence of hypoxaemia (Lindholm et al., 2007), whilst trying to maintain continuous gas exchange (O_2_/CO_2_) between the alveoli, blood and the energy-demanding cell. Redistribution of the cardiac output better maintains the blood flow to organs sensitive to hypoxia, such as the brain and heart, leaving peripheral tissues without sufficient oxygen to metabolize lactate. The increasing accumulation of blood lactate and concomitant progressive acidification is associated with peripherical fatigue in these muscles (Brooks et al., 2022); thus, after a maximal apnoea, PO_2_ may be reduced to as low as 17–22 mmHg while PCO_2_ may be increased to 55–80 mmHg if breath-hold occurs during physical exercise (Schagatay, 2010; Bain et al., 2016). During apnea training the exposure to hypoxia is short and intermittent but much more intense than that suffered by climbers or pilots (Benedek, 2002; Truszczynski et al., 2009); for instance, during a maximal apnea a trained diver could reach as low as 30% oxygen saturation (de Asís Fernández et al., 2019). These combined disturbances in arterial gases trigger a splenic contraction, increasing the levels of circulating red cells in the blood (Richardson, 2009).

Apnoea training has been demonstrated to induce beneficial physiological adaptations that help trained breath-hold divers (BHDs), as well as habitual diving populations, to withstand extreme hypoxaemic/hypercapnic conditions. BHDs are known to employ acute increases in lung volume through the thoracic stretching and glossopharyngeal insufflation (Lindholm, 2005) in order to increase gas storage capacity, i.e., increasing oxygen delivery and also allowing more CO_2_ to be transferred to the lungs during exercice (Schagatay, 2012). Indeed, a previous study has shown that apnoea training increases lung volume and the maximal aerobic capacity (VO_2max_) of swimmers (Lemaitre, 2009). Apnoea training might stimulate anaerobic glycolysis to compensate for the reduction in aerobic metabolism in working muscles, resulting in increased lactate production during breath-hold exercice whilst cycling (Kume, 2013). Furthemore, the overall CO_2_ storage in BHDs has been estimated to be twice that of non-divers (Ferretti, 2001). Thus, the increased total Haemoglobin (Hb) resulting from apnoea training noted above might increase buffering capacity for CO_2_.

Previous reviews suggested that breath-hold divers display beneficial adaptations primarily as a consequence of exposure to training-induced stimuli (Elia et al., 2021), that might contribute to improved sports performance (Lemaître et al., 2010). These adaptations have led researchers to explore the possible use of apnoea training beyond freediving as a means of enhancing performance markers such as: 100, 200 and 400 m swimming time trial (Stavrou et al., 2015; Woorons et al., 2016); 3 km cycling time trial (Bouten et al., 2022); or repeated sprint ability (RSA) (Trincat et al., 2017; Fornasier-Santos et al., 2018; Woorons et al., 2019), defined as an important attribute for athletes competing in high-intensity intermittent sports, such as soccer, basketball, handball, tennis, etc (Bishop and Spencer, 2004).

In line with this ideology, the present article aimed to systematically review the scientific literature and perform a meta-analysis to assess the effects of voluntary apnoea training on markers of aerobic and anaerobic performance, such as maximal aerobic capacity (VO_2max_) and peak blood lactate concentration ([La]_peak_).

## Methods

A systematic review of the literature was performed according to the Preferred Reporting Items for Systematic Reviews and Meta-Analyses (PRISMA) guidelines (Page and Moher, 2017).

### Search strategy

A literature search was conducted on March 2022 by two independent reviewers (DS and JG-R) for the following databases: Web of Science, PubMed and SCOPUS.

Title, abstract and keyword search fields were searched using the following search strategy: “TITLE”: (apnea OR apnoea OR breath hold diving OR free diving OR hypoventilation) NOT (sleep apnea OR sleep apnoea OR obesity hypoventilation syndrome) AND “TITLE, ABSTRACT AND KEYWORDS”: (maximal aerobic capacity OR lactate).

Identification, screening, eligibility assessments and inclusion of studies were performed independently by two reviewers (DS and JG-R) with disagreement settled by a third independent reviewer (FD-F). All records of the literature search were examined by title and abstract to exclude irrelevant records. Studies were selected following the eligibility criteria. Data collected included the author and publication year, sample characteristics, description of intervention and results. If insufficient information was reported, the authors were contacted to confirm additional information about the included studies. Reference lists of selected manuscripts were also screened for any further eligible manuscripts.

### Inclusion criteria

The inclusion criteria were 1) peer-reviewed journal publication; 2) clinical trials; 3) healthy humans; 4) short-term (≤6 weeks), medium-term (7–23 weeks) or long-term (≥24 weeks) effects of apnoea training (Ashton et al., 2020), excluding acute warm-up effects of apnoea; 5) variables included markers of aerobic or anaerobic performance, such as blood lactate and VO_2max_.

### Risk of bias in individual studies

A risk of bias assessment was performed using the Cochrane risk-of-bias appraisal tool (Cochrane Cochrane Handbook for Systematic Reviews of Interventions, 2022). The extracted information enabled appraisal of freedom from risk of bias: “Yes” (low risk), “Unclear” (uncertain risk) or “No” (high risk). We applied this approach to each of seven domains: sequence generation (domain I), allocation concealment used to implement the random sequence (II), blinding of participants and study personnel (IIIa), blinding of outcome assessors (IIIb), incomplete outcome data (IV), selective outcome reporting (V), and other sources of bias (VI). Two authors (DS and FG-M) independently reviewed and scored each study, with disagreement settled by a third independent reviewer (F-DF). Kappa index was calculated to analyze the reliability between the two reviewers before contact with the third reviewer.

### Quality assesment

Strength of the evidence included in this review was determined using the Grading of Recommendations Assessment, Development and Evaluation (GRADE) scale, which shows the overall certainty of the evidence for the outcome being reviewed (Guyatt et al., 2011). The GRADE scale assesses five factors concerning: risk of bias, inconsistency (calculated heterogeneity), indirectness (evidence addresses review question), imprecision (width of confidence intervals [CIs]) and publication bias (funnel plots). These factors lead to a reported score of: “High,” very confident that the true effect lies close to that of the estimate of the effect; “Moderate,” moderately confident in the effect estímate and the true effect is likely to be close to the estimate of the effect but there is a possibility that it is substantially different; “Low,” confidence in the effect estimate is limited and the true effect may be substantially different from the estimate of the effect; or “Very Low,” very little confidence in the effect estímate and the true effect is likely to be substantially different from the estimate of effect.

### Statistical considerations

Meta-analytical procedures were applied to evaluate possible effects of apnoea training (ApT) on the variables studied. Standardised mean difference (SMD) or mean difference (MD) with 95% Confidence Intervals (CI) between ApT and normal breathing (NB) were calculated using a random effects model. It was necessary to contact the authors for further data. Significance for overall effect was set at *p* < 0.05. Heterogeneity of the analysed studies was assessed using an *I*-squared (*I* ^2^) test. Significance level of *I* ^2^ test was set at *p* < 0.05. *I* ^2^ represents the proportion of effects that are due to heterogeneity as opposed to chance (Liberati et al., 2009). Thresholds for low, moderate, and high levels of heterogeneity correspond to *I* ^2^ values of 25, 50, and 75%, respectively. Within the controlled trial studies, a positive effect indicates a larger improvement in the ApT when compared to the NB on VO_2max_ or [La]_peak_, while a negative effect means the opposite. Funnel plots (Egger’s test was not possible) were used to assess publication bias (Sterne and Egger, 2001). All statistical analyses were performed using RevMan 5.3.2 (Nordic Cochrane Centre).

## Results

### Final study selection

**FIGURE 1:**
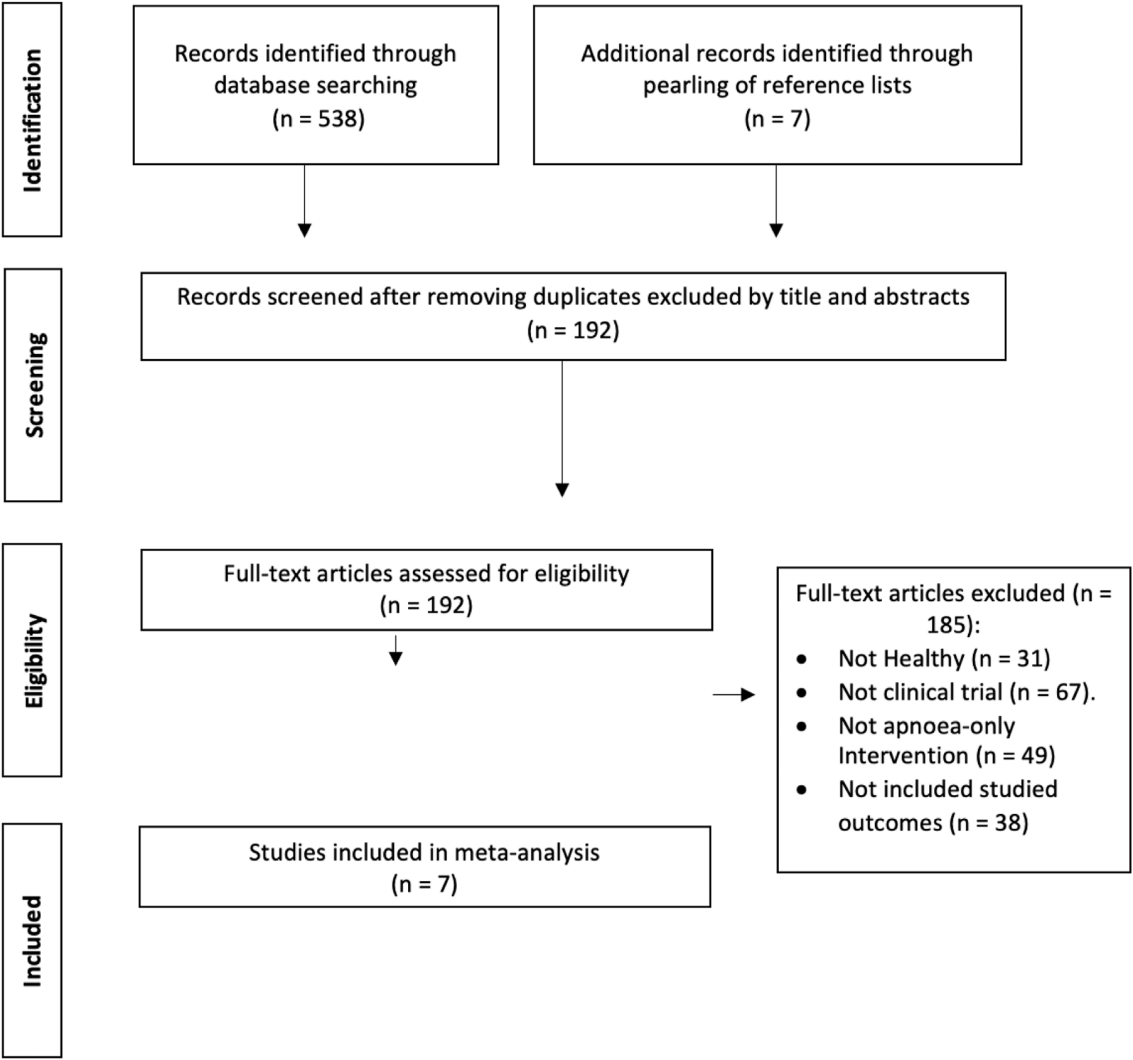
Flow chart of information through the different phases of a systematic review.

538 potential manuscripts were identified following database examination (Figure 1). Snowballing generated seven additional potential manuscripts. After removal of duplicates and elimination of papers based on title and abstract screening, 192 studies remained. Only seven out of 192 studies met the inclusion criteria and were, therefore, included in the meta-analysis (Figure 1).

### Level of evidence and quality of the studies

Using the Kappa index, the overall agreement rate between the two extractors of information prior to correcting discrepant items was 0.841. Risk of bias was identified in several studies, in terms of: lack of sample size estimation; low details on random sequence generation; or, lack of clinical trial registration.

The quality assesment of evidence was judged to be moderate for those variables that were significantly enhanced by ApT over NB. Evidence for other variables was judged to be low quality, indicating that further evidence is required before potential benefits can be excluded. Quality of evidence according to the GRADE system is summarised in Table 1.

**TABLE 1:** Grading of Recommendations Assessment, Development, and Evaluation (GRADE) summary of finding.

Outcomes	Anticipated absolute effects^a^ (95% CI)	No of participants (studies)	Certainty of the evidence (GRADE)
	Risk with ApT		
VO2max	MD 0.89 ml^a^ kg^a^ min-1 higher (1.23 lower to 3.01 higher)	72 (4 RCTs)	⊕⊕⊕○ MODERATE
[La]peak	MD 1.89 mmol^a^ L-1 higher (1.05 higher to 2.73 higher)	86 (5 RCTs)	⊕⊕⊕○ MODERATE

a The risk in the intervention group (and its 95% confidence interval) is based on the assumed risk in the comparison group and the relative effect of the intervention (and its 95% CI). CI: confidence interval; MD: mean difference.

### Characteristics of the participants

Table 2 shows the participant characteristics of the seven studies included in this meta-analysis (total sample size of 127 participants, age 27 ± 9 years old) (Woorons, 2008; Woorons et al., 2019, 2016; Trincat et al., 2017; Fernandez et al., 2018; Fornasier-Santos et al., 2018; Bouten et al., 2022). The total pool of participants was allocated to either ApT (*n* = 64) or NB (*n* = 63). Several different types of recreational athletes were enrolled in the studies, including cyclists (Woorons et al., 2019), rugby-players (Fornasier-Santos et al., 2018), breath-hold divers (Fernandez et al., 2018), triathletes (Woorons et al., 2016), swimmers (Trincat et al., 2017), and runners (Woorons, 2008).

**TABLE 2:** Characteristics of apnoea training (AT) and normal breathing (NB) of included studies. [La]_peak_ peak blood lactate concentration, VO_2max_ maximal aerobic capacity, NS not statistically significant.

Trial (Y/A)	Participants	Level	Intervention	Outcomes	Results
20. Woorons 2016	16 triathletes (12 men and 4 women). Age 33 ± 11 years	No apnoea experience. VO_2max_ = 54± 3 ml*kg^−1^*min^−1^	10 sessions over 5-weeks. ApT (8) vs. NB (8)	[La]_peak_ on 100, 200 and 400 m swim. VO_2max_ on 400 m swim	↑ [La]_peak_ after ApT compared to NB in 100 m (+2.6.±2.2 vs. 0.0 ± 1.1 mM L^−1^; *p* < 0.05), 400 m (2.6.±1.7 vs. 0.2 ± 0.6 mMIL^−1^; *p* < 0.05). In 200-m [La] tended to be greater (1.7.±1.3 vs. 0.4 ± 1.1 mMIL^−1^; *p* = 0.06). NS on VO_2max_ (50.6 ± 11.2 to 52.9 ± 9.3 ml*kg^−1^*min^−1^ in AT vs. 50.1 ± 11.3 to 51.1 ± 9.7 ml*kg^−1^*min^−1^ in NB; *p* > 0.05)
21. Trincat 2016	16 swimmers (9 men, 7 women). Age 15 ± 1 year	No apnoea experience. 9 h swimming plus 3 h conditioning per week	12 sessions over 2-weeks. ApT (8) vs. NB (8)	[La]_peak_ on RSA-swimming	NS [La]_peak_ (8.56 ± 4 to 11.26 ± 4 mM L^−1^ in ApT vs. 9.09 ± 3.2 to 9.96 ± 2 mM L^−1^ in NB; *p* > 0.05)
22. Fornasier-Santos 2018	21 male rugby players. Age 18 ± 1 year	No apnoea experience	8 sessions over 4-weeks ApT (11) vs. NB (10)	[La]_peak_ on RSA-running	NS on [La]_peak_ in ApT vs. NB (13.7 ± 4.3 to 12.6 ± 3.7 mM*L^−1^ vs. 13.0 ± 4.2 to 10.2 ± 3.3 mM*L^−1^).; *p* = 0.25)
23. Woorons 2019	18 male cyclists. Age 34 ± 11 years	No apnoea experience. 10–12 h/week cycling training	6 sessions over 3-weeks ApT (9) vs. NB (9)	[La]_peak_ on RSA-cycling	NS [La]_peak_ in ApT vs. NB (14.0 ± 2.4 to 14.9 ± 3.7 mM*L^−1^ vs. 14.2 ± 3.0 to 13.7 ± 4.1 mM*L^−1^); *p* > 0.05)
24. Bouten et al., 2022	22 physically active males. Age 27 ± 3 years	No apnoea experience. 7 ± 2 h/week physical training	42 sessions over 6-weeks ApT (11) vs. NB (11)	VO_2max_ on incremental running test	NS on VO_2max_ (52 ± 7 to 51 ± 7 ml*kg^−1^*min^−1^ in ApT vs. 50 ± 8 to 48 ± 8 ml*kg^−1^*min^−1^ in NB; *p* = 0.145)
25. Woorons 2008	15 male runners. Age 29 ± 5 years	No apnoea experience. VO_2max_ = 54 ± 3 ml*kg^−1^*min^−1^	12 sessions over 4-weeks ApT (7) vs. NB (8)	VO_2max_ and [La]_peak_ on incremental running test	NS on VO_2max_ (54 ± 2 to 53 ± 3 ml*kg*min^−1^ in ApT vs. 54 ± 5 to 53 ± 5 ml*kg*min^−1^ in NB; *p* > 0.05). NS in [La]_peak_ (15.66 ± 3.9 to 15.03 ± 2.3 mM*L^−1^ in ApT vs. 16.57 ± 3.1 to 14.95 ± 3.1 mM*L^−1^ in NB; *p* > 0.05)
32. De Asís-Fernández 2018	19 male breath-hold divers Age 36 ± 5 years	2 years apnoea experience. VO_2max_ = 53 ± 9 ml*kg^−1^*min^−1^	66 sessions over 22-weeks ApT (10) vs. NB (9)	VO_2max_ on incremental running test	NS in VO_2max_ (47 ± 5 to 47 ± 5 ml*kg^−1^*min^−1^ on ApT vs. 53 ± 9 to 50 ± 8 ml*kg^−1^*min^−1^ on NB; *p* = 0.17)

### Characteristics of the studies selected

The studies included in the review were published between 2008 and 2022. Mean intervention duration was 8 weeks (range 2–22 weeks). Mean training frequency was 3.6 times per week (range 2–7 times per week). The training stimulus of ApT and NB was recorded as overall training stimulus which consists of: multiplying the Rating of Perceived Exertion (RPE) of the global session by its duration (Woorons, 2008) in four studies (Woorons et al., 2019, 2016; Trincat et al., 2017; Fornasier-Santos et al., 2018); as % oxygen saturation (SpO_2_) in four studies (Woorons et al., 2019, 2016; Trincat et al., 2017; Fornasier-Santos et al., 2018); as RPE in four studies (Woorons et al., 2019, 2016; Trincat et al., 2017; Fernandez et al., 2018); as HR in three studies (Woorons et al., 2016; Trincat et al., 2017; Fornasier-Santos et al., 2018) and as [La]_peak_ in three studies (Woorons et al., 2016; Trincat et al., 2017; Fornasier-Santos et al., 2018). Results consistently confirmed that oxygen saturation was lower in ApT than NB, but that each of the four papers reported these data in different ways. Tables 2, 3 show the characteristics of the apnoea training interventions for the seven studies included in this review.

**TABLE 3:** Apnoea training interventions of included studies. DNF dynamic apnoea no fins DYN dynamic apnoea with fins, HLV_DNF, DYN, STA_ dynamic apnoea no fins, dynamic apnoea fins and static apnoea at high lung volumes, LLV_cycling_ apnoea cycling at low lung volumes, LLV_run_ apnoea running at low lung volumes, LLV_swim_ apnoea swimming at low lung volumes, r recovery between repetitions, R recovery between sets.

Trial (Y/A)	Weeks	Session/weeks	ApT intervention	Sets	Reps	Load	r	R	ApT (%SpO2)						NB (%SpO2)			
24. Bouten et al., 2022	6	7	HLV STA	1	5	Max	30s		—						—			
23.Woorons 2019	3	2	LLV cycling	2–3	6–8	6 s	24 s	3 min	mean 87%						mean 95%			
22.Fornasier-Santos 2018	4	2	LLV run	2–3	8	40 m	Start each 30 s	3 min	>96%	93%–96%	89%–92%	85%–88%	<85%	>96%	93%–96%	89%–92%	85%–88%	<85%
									5 s	108 s	169 s	138 s	18 s	126 s	302 s	11 s	0 s	0 s
32.De Asís-Fernández 2018	22	3	HLV DNF, DYN, STA	2	3	Max	180 s	10 min	—						—			
20.Woorons 2016	5	2	LLV swim	1	10–12	25 m	Start each 30 s	—	—	92%–94%	88%–91%	<88%	—	—	92%–94%	88%–91%	<88%	—
									—	118 s	89 s	55 s	—	—	1 s	0 s	0 s	—
21.Trincat 2016	2	6	LLV swim	2	16	15 m	Start each 30 s	20 min	>98%	95%–98%	92%–95%	90%–92%	<90%	>98%	95%–98%	92%–95%	90%–92%	<90%
									102 s	324 s	56 s	24 s	40 s	216 s	402 s	6 s	2 s	0 s
25.Woorons 2008	4	3	LLV run	4	5 min	45 s	15 s	1 min	—						—			

### Anaerobic exercise performance

A total of five studies analyzed the effects of ApT on anaerobic exercise performance (Woorons, 2008; Woorons et al., 2019, 2016; Trincat et al., 2017; Fornasier-Santos et al., 2018), with [La]_peak_ the only variable reported. The overall quality of evidence was judged to be moderate evidence for [La]_peak_ (Table 1).

**FIGURE 2:**
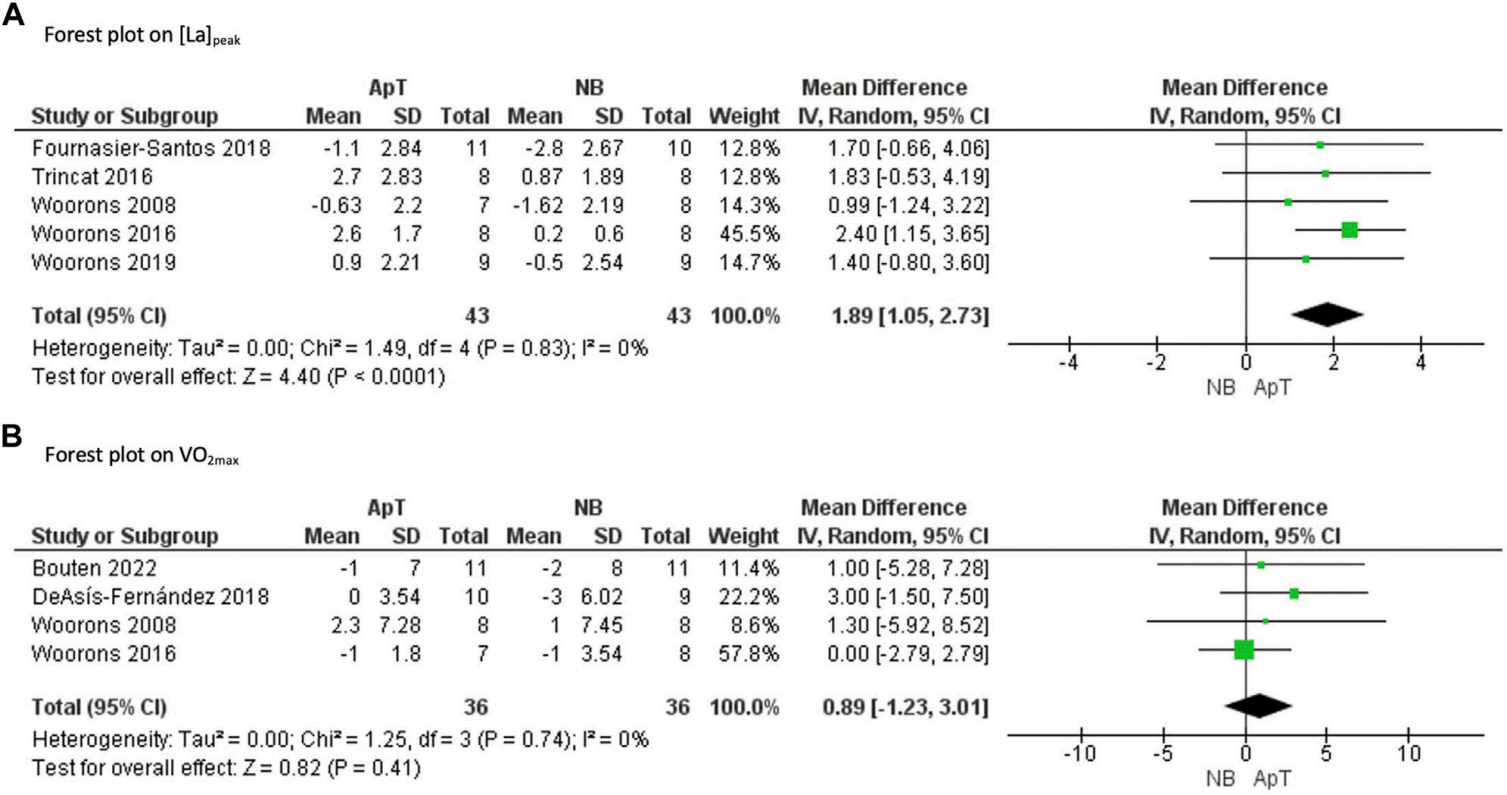
Forest plot of the effects of apnoea training (ApT) and Normal Breathing (NB) on [La]_peak_ peak blood lactate concentration **(A)** and VO_2max_ maximal aerobic capacity **(B)**. CI confidence interval, SD standard deviation, IV weighted mean difference.

The [La]_peak_ increased to a greater extent following ApT when compared to NB (MD = 1.89 mmol*L^−1^ [95% CI 1.05, 2.73], Z = 4.40, *p* < 0.0001). The *I* ^2^ test showed a non-significant heterogeneity among the included studies (*I* ^2^ = 0%, *p* = 0.83). These results are displayed in Figure 2A.

### Aerobic exercise performance

A total of four studies analyzed the effects of ApT on aerobic exercise performance (Woorons, 2008; Woorons et al., 2016; Fernandez et al., 2018; Bouten et al., 2022), with VO_2max_ the only variable reported. The overall quality of evidence was judged to be moderate evidence on VO_2max_ (Table 1).

Results show that there were no differences in changes to VO_2max_ following ApT compared with NB (MD = 0.89 ml*kg*min^−1^ [95% CI −1.23, 3.01], Z = 0.82, *p* = 0.41). The *I* ^2^ test showed a non-significant heterogeneity among the included studies (*I* ^2^ = 0%, *p* = 0.74). These results are displayed in Figure 2B.

## Discussion

This is the first review to systematically analyze the longitudinal effects of apnoea training on aerobic and anaerobic performance. The main finding of this systematic review was that longitudinal (2–22 weeks) apnoea training (ApT) increases peak blood lactate concentration to a greater extent than control interventions using normal breathing (NB) in recreational athletes. However, we found no significant increase for maximal aerobic capacity after longitudinal ApT.

In terms of rationale for aerobic performance, cross-sectional data show that trained breath-hold divers exhibit higher lung and splenic volumes (Schagatay, 2012), greater oxygen and carbon dioxide stores in tissues (Liner, 1994), intensified oxygen conserving mechanisms (Costalat, 2015), and blunted ventilatory chemosensitivity (Roecker et al., 2014) compared with non-diving controls. However, the lack of controlled clinical trials previously made it difficult to evaluate the strength of supporting evidence. Although these studies reported some promising data suggesting potential beneficial effects of ApT for exercise performance, methodological limitations must be considered. The physiological adaptations acquired by the BHDs goes beyond an isolated breath-hold stimulus (i.e. stretching, endurance, inspiratory muscle training, swimming, glossopharyngeal insufflation, etc.), so such other interventions are likely confound the effectiveness of ApT. As the effects mentioned above are advantageous in several sports, we hypothesised that ApT may be an effective alternative to hypobaric or normobaric hypoxia to increase aerobic and/or anaerobic performance.

Oxygen supply and transport capacity to the working mucles are important determinants of aerobic performance (Brugniaux, 2006), usually measured by VO_2max._ Since most of the oxygen in the blood is bound to Hb, this oxygen transport capacity of the body is largely determined by the amount of Hb. Previous studies (Joulia, 2002; Prommer, 2007; Richardson, 2008) have shown a marked correlation between total blood hemoglobin (Hb) and VO_2max_, furthermore showing that lowering Hb (e.g. after blood donation) decreases oxygen uptake, whereas increasing Hb enhances VO_2max_. In their study, Schmidt and Prommer (Prommer, 2007) have shown an increase of 1 g in Hb corresponding to an increase of 4 ml kg^−1^ min^−1^ in VO_2max_. In this context, ApT has been proposed as a new hypoxic training method, since serum erythropoietin (EPO) levels increase in a dose-dependent manner in response to reductions in blood oxygenation (Levine, 1997). Hypoxic training effects depend mainly on the conditions of the hypoxia exposure—mainly the severity of hypoxia and length of exposure (de Asís Fernández et al., 2019). Accordingly, a variety of methods have been developed to augment endogenous EPO. For example, the ‘‘live high, train low” method is widely used by endurance athletes (Levine, 1997), consisting of combining resting exposure to hypoxia with normoxic training. Nevertheless, it is possible to generate hypoxaemia without the many challenges of being placed in a hypoxic environment, ie by voluntarily reducing the breathing frequency. It should be noted that hypoxia during ApT can be severe, although it is only for a very short time, far shorter than more chronic exposures to hypobaric hypoxia such as seen in mountaineers (Benedek, 2002).

In the studies included in this review (Woorons, 2008; Woorons et al., 2019, 2016; Trincat et al., 2017; Fernandez et al., 2018; Fornasier-Santos et al., 2018; Bouten et al., 2022), it was reported that athletes could train under hypoxic conditions at sea level through apnoea training at low lung volumes (LLV, i.e., “end-expiratory” breath-hold). During such exercise with ApT at LLV, arterial oxygen saturation dropped to severe hypoxaemia. Conversely, when athletes applied ApT at higher lung volumes (–HLV, i.e., “end-inspiratory” breath-hold) no hypoxic effect occurred.

In addition, the hypoxic stimulus increases long-term splenic volume and contractility, increasing the red blood cell reservoir in hypoxic situations, thus acutely improving oxygen delivery in these conditions. Such an effect would be an effective warm-up to enhance red blood cell volume, however these improvements disappears within 10 min after the last apnoea (Schagatay et al., 2005), which may limit application for longer lasting activities.

Another factor that will influence aerobic performance will be respiratory function. Trained BHDs typically exhibit high lung volumes (Andersson, 2004; Schagatay, 2012; Fernandez et al., 2018), range 5.9–7.3 L of VC, that are higher than in those unaccustomed to ApT. Usually, BHDs training includes concomitant training such as stretching, aerobic training, glossopharyngeal insufflation, or inspiratory muscle training. Such training has shown positive effects on increasing VC (Enright and Unnithan, 2011; Khosravi et al., 2013), but it is not possible to differentiate the effect of ApT on VC through such observational studies. Based on our findings, it is recommended that future studies evaluating the effect of ApT on VC distinguish the effects produced on BHDs (near their physiological limit) from participants without apnoea experience, who would have a greater scope for improvement.

Previous reviews consulted (Lemaitre, 2009; Schagatay, 2010; Elia et al., 2021) all agree that ApT might be an effective alternative to increase hematocrit, EPO, Hb and lung volume; all of them predictors of aerobic fitness. However, in stark contrast, the randomised control trials included in this review (Woorons, 2008; Woorons et al., 2016; Fernandez et al., 2018; Bouten et al., 2022) suggest no effects of ApT on aerobic performance measured by VO_2max_. It is of course acknowledged that there are many other parameters of endurance performance, beyond VO_2max_, that are arguably more ecologically valid and these were not assessed in the reported studies. Furthermore, factors such as accelerated recovery processes or muscle buffering capacity could also be important for performance (Bishop et al., 2004) and again were not assessed.

Accordingly, the meta-analysis results might suggest that improvements in anaerobic capacity may be more likely to have influenced the greater improvements in performance following ApT (Stavrou et al., 2015), though other factors cannot yet be excluded. A total of five clinical trials analyzed the effects of ApT on anaerobic exercise performance (Woorons, 2008; Woorons et al., 2019, 2016; Trincat et al., 2017; Fornasier-Santos et al., 2018). These studies showed that ApT seems to be advantageous for improving anaerobic glycolysis. For instance, results showed improved performance of short actions at maximal or near to maximal intensity, separated by short recovery periods and for a relatively long period of time (between 1 and 4 h), which are reflective of sport-specific demands for athletes competing in high-intensity intermittent sports, such as soccer, basketball, handball, tennis, etc (Bishop et al., 2001, *p*. 2004). During such repeated sprints, the majority of ATP resynthesis comes from anaerobic metabolism, but with progressive sprint number there is a decline in energy provided by the anaerobic system and consequently, despite increased aerobic contribution, muscle power cannot be maintained, with a concomittant decline in performance (Gaitanos et al., 1993).

The meta-analysis revealed increases in [La]_peak_, without benefits for aerobic markers, supporting the underpinning mechanisms are more related to improvements in anaerobic capacity. Such mechanisms may include increases in glycogen and phosphocreatine availability, enzymatic activity, buffering capacity, or even improved “tolerance” of the exercise-induced acidosis (Bishop et al., 2011, *p*. 2011). Training adaptations of anaerobic glycolysis may include both increased maximum rates of lactate production and improved muscle-buffering capacity which leads to increase lactate clearance and minimize pH disturbance in muscle (Sharp et al., 1986). The improved [La]_peak_ supports this, but cannot differentiate effects further as net blood lactate values reflect the continuous balance of muscle lactate production and clearance (Brooks, 2018) and both processes may have been affected.

It is necessary to acknowledge several limitations to this systematic review. These include the wide variety of different protocols for apnoea training interventions, such as differences in biomechanics (run, cycling, and swim); training volume and frequency; lung volume (low or high); apnoea duration (6 s to maximal); and recovery (4 s–20 min). For future perspectives, it is recommended that researchers carry-out clinical trials analysing isolated apnoea training effects to find the optimal characteristics of the training method (e.g., training volume, lung volume, apnoea duration or recovery time), as well as using more ecologically valid markers of aerobic performance.

## Practical applications

This study highlights the importance that ApT might be an effective intervention to enhace anaerobic performance by increasing peak blood lactate concentration in athletes who practice high-intensity intermittent sports such as, racquet sports, mixed martial arts, or team sports. It may also be of interest for high-intensity continuous sports such as sprinting in swimming, athletics, or cycling.

A great variability has been found in the training methods, for example in the duration of apnoea efforts. Some training methods use maximal apnoeas with long rest periods (Fernandez et al., 2018) while other studies use short-repeated apnoeas with short rest periods (Fornasier-Santos et al., 2018). Repeated apnoea training seems to be the most optimal method for use in athletes unfamiliar with apnoea training, as it is necessary to train an individual’s psychological tolerance of maximal apnoea.

It is possible to generate severe hypoxaemia, increased blood lactate concentration and a greater glycolytic activity without the costs and challenges of being placed in a hypoxic environment, by using apnoea training at low lung volumes, eliciting faster hypoxaemia.

Apnoea training, as with blood flow restriction training, could be a suitable intervention when athletes are injured and not training, or doing rehabilitation with limited high-intensity work.

## Data Availability

The original contributions presented in the study are included in the article/supplementary material, further inquiries can be directed to the corresponding author.

## Conclusion

The meta-analysis findings of improved [La]_peak_, without benefits for aerobic markers, suggests that the underpinning mechanisms may lie more with improvements in anaerobic capacity. Such improvements may include increases in glycogen and phosphocreatine availability, increased enzymatic activity, buffering capacity, or even improved “tolerance” of the exercise-induced acidosis.
